# *B-BOX* genes: genome-wide identification, evolution and their contribution to pollen growth in pear (*Pyrus bretschneideri* Rehd.)

**DOI:** 10.1186/s12870-017-1105-4

**Published:** 2017-09-19

**Authors:** Yunpeng Cao, Yahui Han, Dandan Meng, Dahui Li, Chunyan Jiao, Qing Jin, Yi Lin, Yongping Cai

**Affiliations:** 1 School of Life Sciences, Anhui Agricultural University, Hefei, 230036 China; 2 State Key Laboratory of Tea Plant Biology and Utilization, Anhui Agricultural University, Hefei, 230036 China

**Keywords:** *B-BOX*, Systematic analysis, Pear, Relative gene expression

## Abstract

**Background:**

The B-BOX (BBX) proteins have important functions in regulating plant growth and development. In plants, the *BBX* gene family has been identified in several plants, such as rice, Arabidopsis and tomato. However, there still lack a genome-wide survey of *BBX* genes in pear.

**Results:**

In the present study, a total of 25 *BBX* genes were identified in pear (*Pyrus bretschneideri* Rehd.). Subsequently, phylogenetic relationship, gene structure, gene duplication, transcriptome data and qRT-PCR were conducted on these *BBX* gene members. The transcript analysis revealed that twelve *PbBBX* genes (48%) were specifically expressed in pear pollen tubes. Furthermore, qRT-PCR analysis indicated that both *PbBBX4* and *PbBBX13* have potential role in pear fruit development, while *PbBBX5* should be involved in the senescence of pear pollen tube.

**Conclusions:**

This study provided a genome-wide survey of *BBX* gene family in pear, and highlighted its roles in both pear fruits and pollen tubes. The results will be useful in improving our understanding of the complexity of *BBX* gene family and functional characteristics of its members in future study.

**Electronic supplementary material:**

The online version of this article (10.1186/s12870-017-1105-4) contains supplementary material, which is available to authorized users.

## Background

Zinc-finger protein is one of the important transcription factors, which play important roles in plant growth, development and response to environmental changes. The zinc-finger protein whose three-dimensional is stabilized by binding zinc ions [1], could interact with DNA, RNA, and proteins involved in plant cell life activities [2]. *B-BOX* gene family belongs to the zinc-finger protein family. In addition to the conserved B-BOX domain, some of the B-BOX members contain other family-specific domains, such as CCT (CONSTANS, CO-like and TOC1) domain. The *B-BOX* gene family could be divided into five subfamilies according to the number of B-BOX domains or the CCT domain they contained [2]. After the first identification of the B-BOX member from *Xenopus laevis* [3], its ortholog (CONSTANS: CO) in plant was cloned from *Arabidoosis thaliana*, with function in the photoperiod regulation of plant flowering time [4]. Subsequent researches further revealed that the B-BOX transcription factors in plants played very pivotal roles in mediating various life activities, such as seed germination [5], flowering [6], shade avoidance response [7], biological or abiotic stress response [8] and plant hormone signal transduction [9]. Recent studies have shown that as a negative regulator in brassinosteroid signaling pathways, *BBX20* (*AtBZS1*) from *A. thaliana* could attach to E2 by recognition of COP1 (Constitutively Photomorphogenic 1), a key factor in light signal transduction, then for degradation by 26S proteasomes [9]. Gangappa et al. (2013) stated that *AtBBX25* is involved in the negative regulation of plant photo-morphogenesis by forming a dimer with HY5 (Protein long hypocotyl 5) and suppressing its function [10]. Studies in apples found that *MdBBX22* (*MdCOL11*) involved in the UV-B-induced synthesis of the anthocyanin synthesis [11]. Based on these results, it was implied that *B-BOX* gene members might be associated with COP1-HY5-mediated optical signal transduction pathway, and further involved in plant light morphogenesis, regulating flowering, secondary metabolite synthesis and regulation of a variety of life activities.

It has been proved that some transcription factor family should play an important role in the development of pear fruits, such as MYB and heat shock factor gene family [12, 13]. Two MYB family members, *PbMYB25* and *PbMYB5*2 were found to be the candidate genes involved in the regulation of lignin synthesis within pear fruits [12]. Although much knowledge on the function of *B-BOX* gene family, such as responses to biotic and abiotic stresses and involvement in light signal transduction pathway, has been advanced [14–16], there is less research on their roles in pear pollen and fruit development. The completed pear genome sequencing [17] provided useful information for comprehensive analysis of the pear *BBX* gene family. In this study, 25 non-redundant members were identified in the pear *BBX* family. Subsequently, the detailed phylogenetic and expression pattern analyses of these *BBX* genes were carried out. The present results will be useful for further functional characterization of *BBX* genes in pear.

## Results

### Identification of *BBX* genes in pear

To obtain BBX proteins in pear genome, the published Arabidopsis BBX proteins were employed as a query to search against the local pear genome database by using DNAtools software. After removing the redundant and repeated sequences, a total of 25 putative BBX protein sequences were confirmed in pear. For the sake of consistency, these *BBX* genes were sequentially named after *PbBBX1* to *PbBBX25*. The detailed information on the gene identifier, chromosome location, protein structure and the characteristics of the corresponding PbBBX proteins were listed in Table 1. The length of the amino acid sequence sequences ranged from 142 (PbBBX23) to 859 (PbBBX7). The pear *BBX* genes encode proteins with predicted theoretical isoelectric points of 4.48–9.02 and molecular weights from 15.63900 (PbBBX23) to 93.60447 (PbBBX7) kDa (Table 1).

**Table 1:** The detailed information of *PbBBX* members

Gene	Gene identifier	5′ End	3′ End	Chr	AA	pls	MW	Domains	Structure
*PbBBX1*	Pbr016562.1	17,929,143	17,931,284	17	397	5.41	43.92956	2BBX + CCT	I
*PbBBX2*	Pbr023570.1	13,087,654	13,089,051	16	424	7.87	45.89379	2BBX + CCT	I
*PbBBX3*	Pbr019957.1	5,731,876	5,732,987	15	340	5.84	37.97752	2BBX + CCT	I
*PbBBX4*	Pbr036464.1	16,593,377	16,594,915	8	340	5.95	37.81046	2BBX + CCT	I
*PbBBX5*	Pbr040252.1	20,855,304	20,857,692	17	490	6.54	53.16922	2BBX + CCT	II
*PbBBX6*	Pbr022786.1	1,461,782	1,463,416	3	379	5.49	41.19483	2BBX + CCT	II
*PbBBX7*	Pbr026954.1	6,184,619	6,192,599	13	859	5.2	93.60447	2BBX + CCT	II
*PbBBX8*	Pbr038936.1	15,996,677	15,998,414	14	447	5.06	50.13728	1BBX + CCT	III
*PbBBX9*	Pbr020281.1	3,789,123	3,790,874	6	459	5.26	51.07542	1BBX + CCT	III
*PbBBX10*	Pbr013295.1	21,079,074	21,081,045	3	453	5.23	50.07548	1BBX + CCT	III
*PbBBX11*	Pbr028831.1	2,106,467	2,108,184	13	454	5.45	50.59912	1BBX + CCT	III
*PbBBX12*	Pbr022361.1	24,883,049	24,886,946	10	208	5.88	22.95886	2BBX	IV
*PbBBX13*	Pbr038976.1	52,556	56,089	5	199	5.68	22.02697	2BBX	IV
*PbBBX14*	Pbr042773.1	19,278,411	19,280,758	15	185	7.05	20.53247	2BBX	IV
*PbBBX15*	Pbr019591.1	648,141	649,024	5	222	6.34	24.86486	2BBX	IV
*PbBBX16*	Pbr015820.1	26,044,737	26,045,718	10	224	5.92	25.04003	2BBX	IV
*PbBBX17*	Pbr005884.1	2,824,859	2,826,234	15	302	6.82	33.21935	2BBX	IV
*PbBBX18*	Pbr020473.1	1,098,702	1,100,900	11	288	5.47	30.95676	2BBX	IV
*PbBBX19*	Pbr032616.1	3,755,943	3,757,523	9	243	5.29	26.51099	2BBX	IV
*PbBBX20*	Pbr034751.1	1,633,105	1,635,052	17	242	5.09	26.64001	2BBX	IV
*PbBBX21*	Pbr033352.1	41,552,552	41,553,827	15	246	4.55	26.4555	1BBX	V
*PbBBX22*	Pbr011255.1	22,264,853	22,266,136	17	249	4.48	26.96689	1BBX	V
*PbBBX23*	Pbr000255.1	26,681,876	26,682,798	5	142	4.51	15.639	1BBX	V
*PbBBX24*	Pbr022252.1	14,907,160	14,908,704	17	270	8.93	29.41226	1BBX	V
*PbBBX25*	Pbr031832.1	19,005,388	19,007,067	15	271	9.02	29.72965	1BBX	V

*Chr* chromosome, *AA* number of amino acid, *pIs* theoretical isoelectric point, *MW* molecular weight, *KDa* kilodalton

### Protein sequence and phylogenetic analysis of the pear *BBX* gene family

**Fig. 1:**
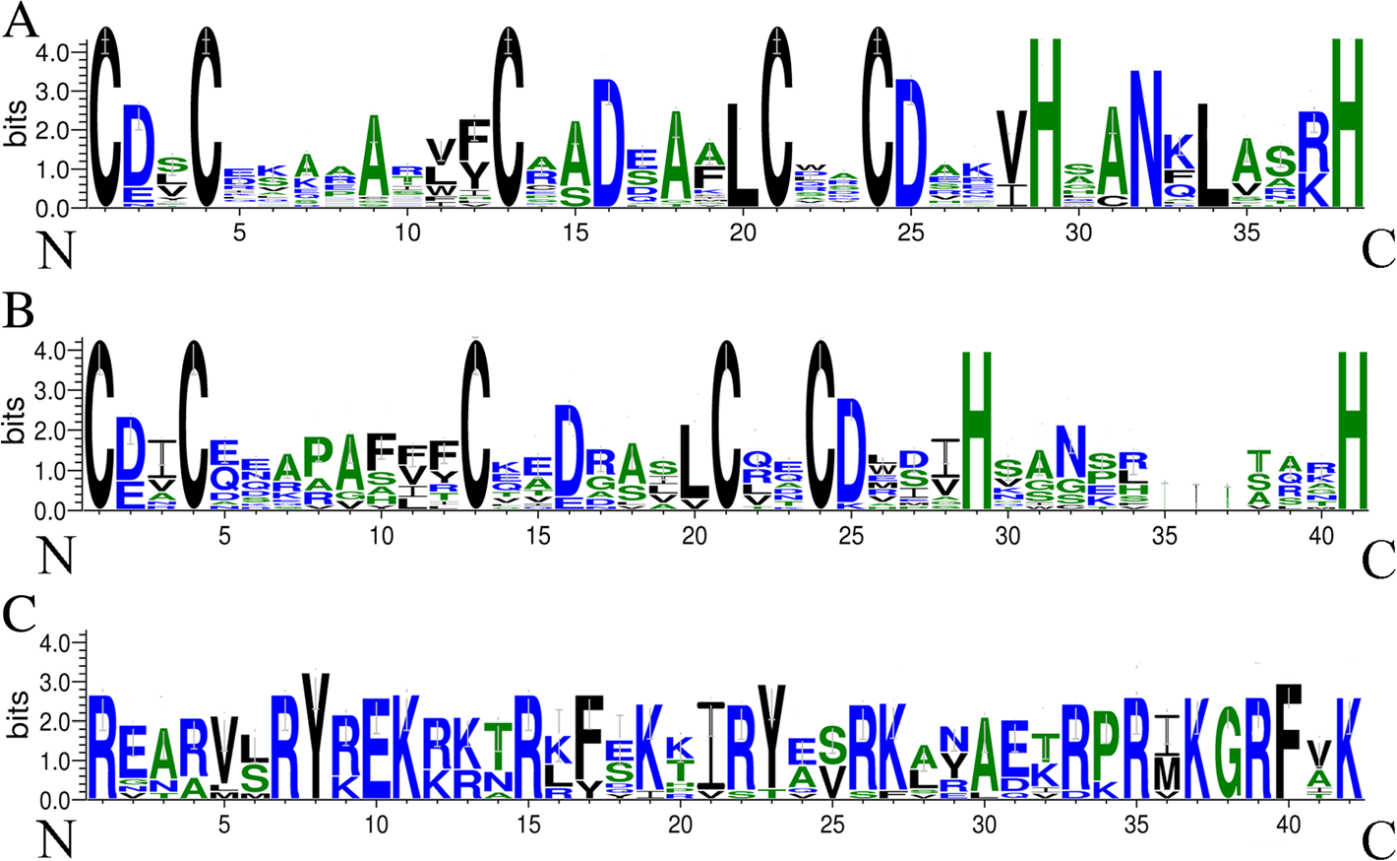
Domain composition of PbBBX proteins. **a**, **b** and **c** represent the protein alignment of the B-BOX 1, B-BOX 2 and CCT domain, respectively. The x-axis indicates the conserved sequences of the domain. The height of each letter indicates the conservation of each residue across all proteins. The y-axis is a scale of the relative entropy, which reflects the conservation rate of each amino acid

**Fig. 2:**
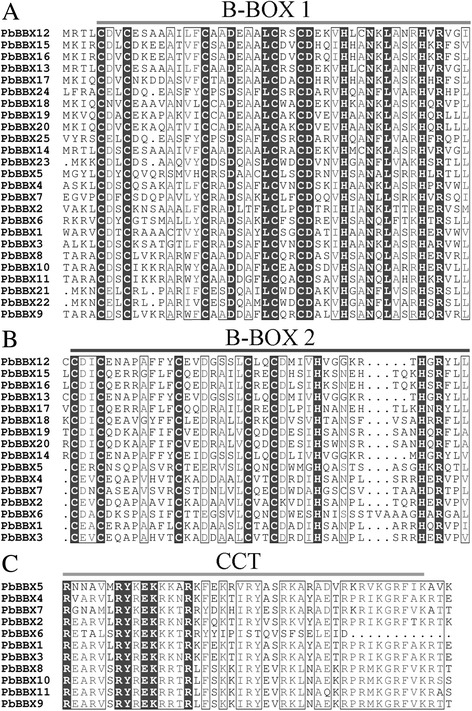
Multiple sequence alignments of the domains of the PbBBXs. Multiple sequence alignments of the B-box 1 (**a**), B-box 2 (**b**) and CCT (**c**) domains are shown. The identical conserved amino acids were represented by black shaded

The identified PbBBX proteins showed a wide variation of molecular length ranged from 142 to 859 amino acids. Out of 25 PbBBXs, seven were found to contain a conserved CCT domain and two B-BOX domains. Four and five members contained one B-BOX plus a CCT domain, or only one B-BOX domain, respectively, with the remaining nine members containing two B-BOX domains (Table 1). The conserved structures of PbBBX members, were found with B-Box 1 sequence (CDXCXXXXAXVYCXADEAALCXXCDXXVHXANKLAXRHXH, X represents any amino acid) and B-Box 2 (CDICXXXXAXXXCXXDXAXLCXXCDXXVHXXXXXXHXRXXL) (Fig. 1). Additionally, the CCT domain was highly conserved among the PbBBXs (Fig. 1). The logos of these domains, including B-BOX1, B-BOX2 and CCT domain, were illustrated in Fig. 1, as well as the correspondence positions shown in Fig. 2.

**Fig. 3:**
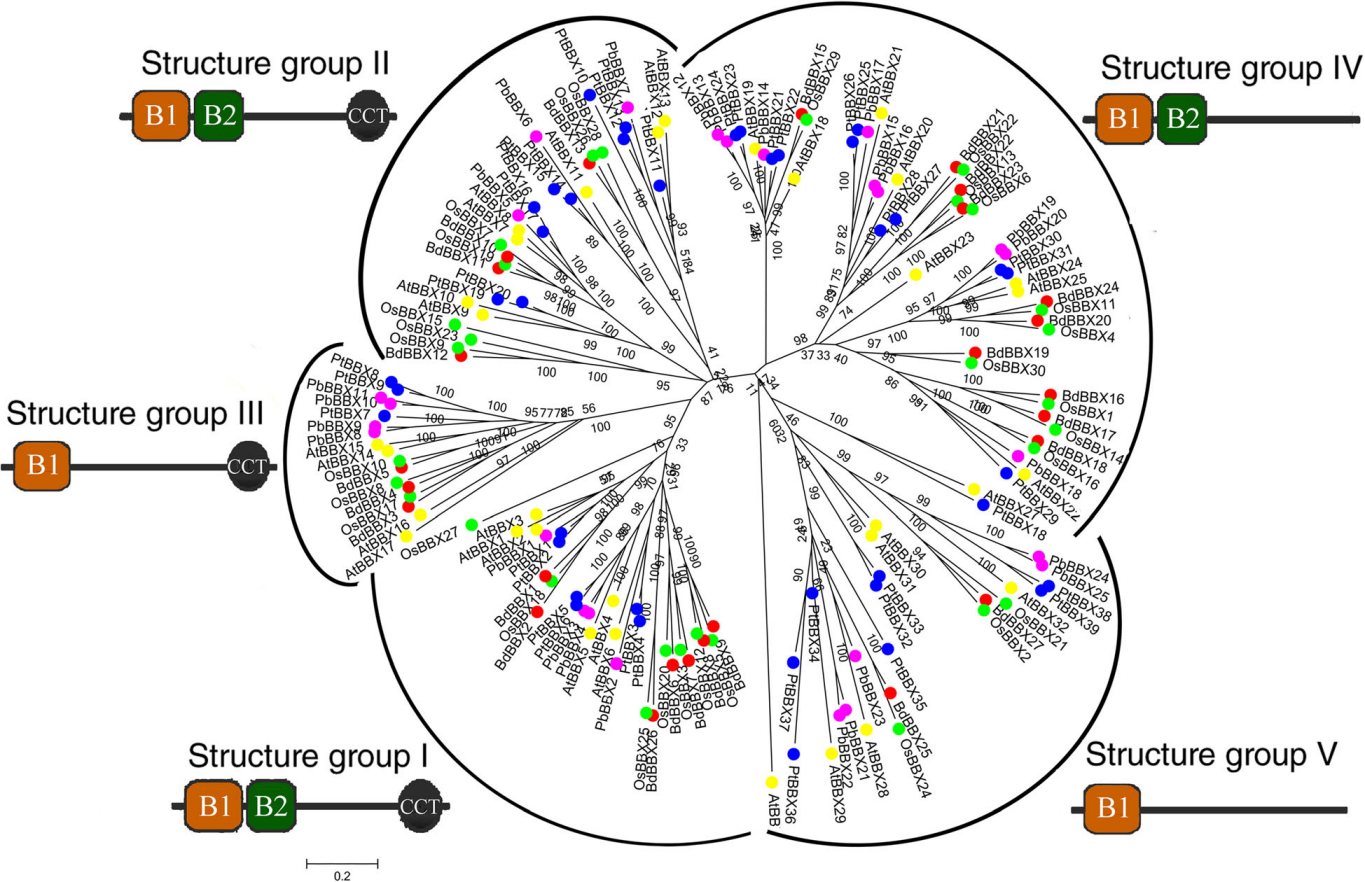
Phylogenetic analysis of *BBX* genes in pear, *Brachypodium distachyon*, *Oryza sativa*, *A. thaliana* and *Populus trichocarpa*. The scale bar represents 0.1 amino acid substitutions per site. In addition, B-box domain type 1, B-box domain type 2 and CCT domain were represented by B1, B2 and CCT, respectively

To gain further insights into the phylogenetic relationship and divergence of the *BBX* family, the phylogenic tree, including BBXs from *Brachypodium distachyon*, *Oryza sativa*, *A. thaliana*, *Populus trichocarpa* and pear, was constructed. Based on the phylogenetic analysis, this tree could be divided into five clades, and consistent with the previous studies [2, 18]. As shown in Fig. 3, most BBX members from poplar, Arabidopsis and pear were more closely than pear and *Oryza sativa*, *Brachypodium distachyon*. Among them, the members from clades I, II, III, contained two B-BOX domains plus a CCT domain, implying that these genes contained CCT-domain might play a crucial role in the control of flowering [4, 19]. On the contrary, the members from clade VI and clade V lacked CCT-domain and only contained one or two B-BOX domain. Previous studies have shown that the B-BOX domains (CX_2_CX_8_CX_7_CX_2_CX_4_HX_8_H**)** in the N-terminal region, and the conserved Cysteine (C) and Histidine (H) residues in B-BOX domain are predicted to be crucial for BBX protein–protein [2]. Interestingly, the Cysteine (C) and Histidine (H) residues was also found to be conserved at B-BOX domain in the C-terminal region of the BBX members from clades I, II and IV, respectively (Fig. 1). In summary, the structure analyses of theses B-BOX proteins were basically consistent with the phylogenetic relationship.

### Gene structure and gene duplication

**Fig. 4:**
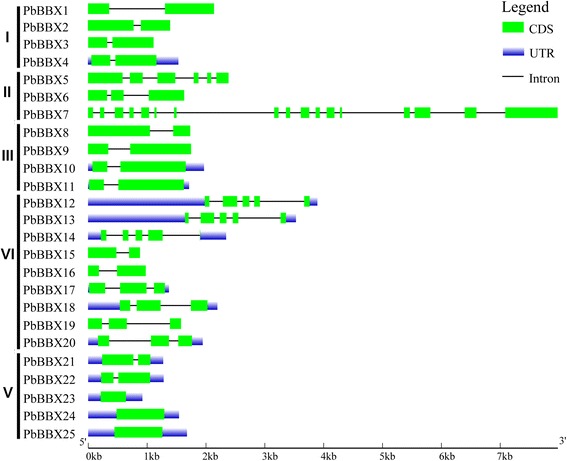
Exon-intron structure of the *PbBBX* family generated from GSDS online website. Legend is at the top right of the Figure. The scale represents the length of the DNA sequence

The previous studies implied that gene structural diversity can lead to the evolution of multi-gene families. To better characterize and understand the structural diversity of the *PbBBX* genes, gene exon-intron analysis was carried out (Fig. 4). As shown in Fig. 4, the number of exons was ranged from 1 to 17, with *PbBBX7* containing the highest amounts of exons (17) among the *PbBBXs*, 12 of *PbBBXs* containing two exons, and 3 only one exon, respectively. Additionally, pear *BBX* genes were clustered in the same clade with the highly similar exon-intron structure, for example, eight *PbBBX*s within the clades I and III (containing two exons), and most members belonging to clade IV (having three exons). Likewise, three genes in clade V only contained one exon, except for *PbBBX24* and *PbBBX25*. These results deduced that exon-loss or -gain had occurred during the evolution of the *PbBBX* gene family and resulted in the functional divergence among the closely related *PbBBXs* (Fig. 4).

**Fig. 5:**
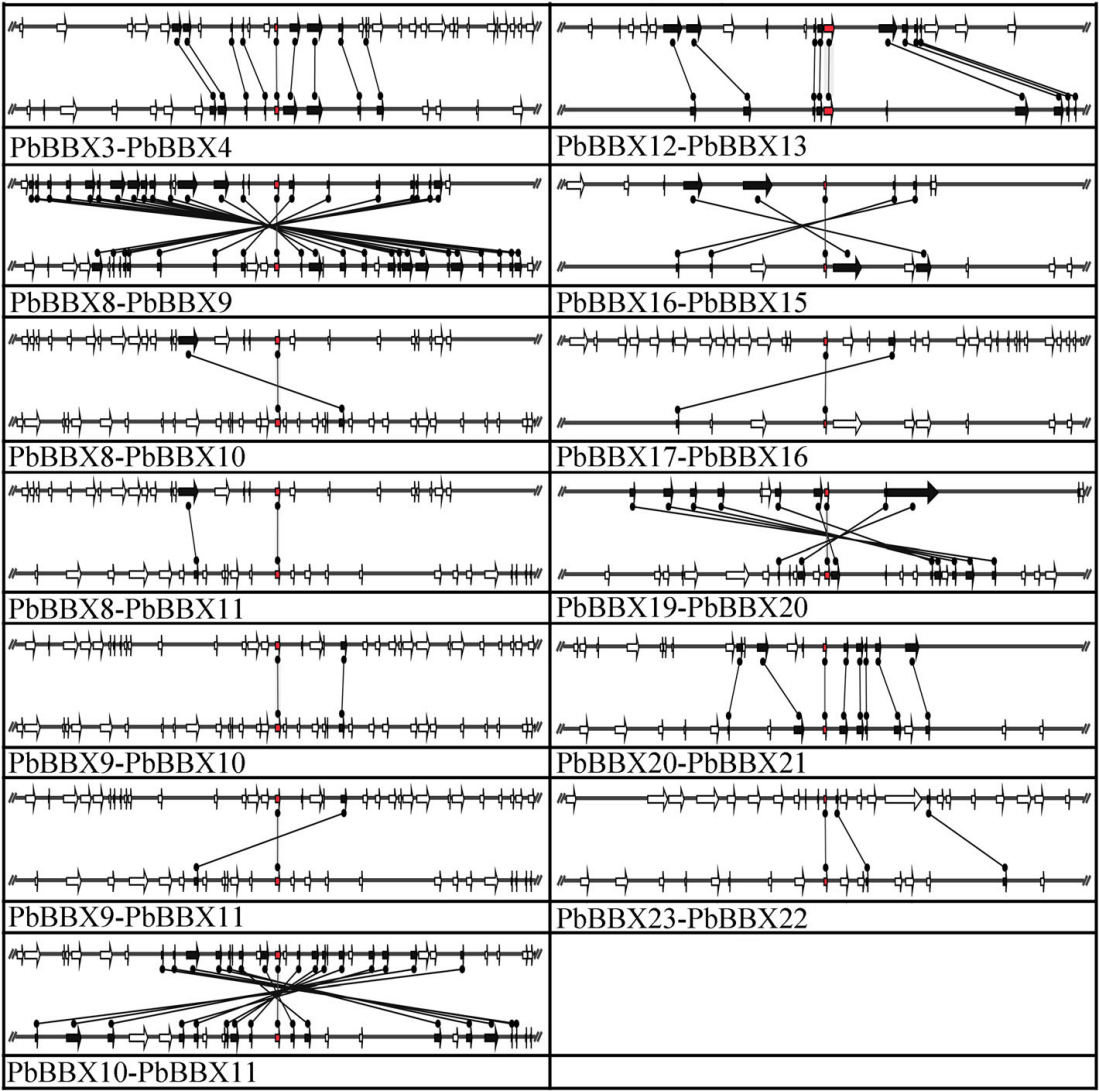
Segmental duplication between members of the *BBX* gene family in pear. The map shows the 100 kb region on each side flanking of the BBX genes. The conserved gene pairs among the segments are connected with bands. Gene and its transcriptional orientation were represented by broad line with arrowhead

Up to data, the information about the expansion events of the *BBX* gene family in pear was still unclear. To further reveal how *PbBBX* genes were evolved, the chromosomal location and gene duplication events of *PbBBX* genes were investigated. The chromosome locations and distributions of 25 *PbBBX* genes were found among the 12 pear chromosomes (total of 17 chromosomes) (Additional file 1: Figure S1). Among them, the chromosomes 15 and 17 both contained highest number of *PbBBX* genes (5); followed by chromosome 5 contained three genes; chromosomes 3 and 10 both had two genes; while the chromosomes 6, 8, 9, 11, 13, 14 and 16 only had one genes. Gene family expansion was usually achieved by tandem duplication and segmental duplication. In present study, we did not identify any of tandem duplication pairs. However, 13 segmental duplication gene pairs were found in pear genome by using MCScanX software (Fig. 5). Subsequently, the divergence time between these gene pairs was calculated with the period varied from 6.15 to 253.08 million years (Mya) (Additional file 2: Table S1).

To determine the selection pressure in duplication of *PbBBX* genes, the non-synonymous (Ka)/synonymous (Ks) values were calculated for the 13 gene pairs. The Ka/Ks values of all *PbBBX* gene pairs were less than 1.0 (Additional file 2: Table S1), indicating that they were under strong purifying selective during their evolution and a conserved evolutionary pattern was shared among *BBX* genes.

### Expression patterns of pear *BBX* genes

The pollen germination and pollen tube growth in many higher plants have been known to play a crucial role in sexual reproduction. Previous studies suggested that pollen tube via tip-growth rapidly extended and then underwent senescence within 15 h (P4: stopped-growth pollen tubes) in vitro [20]. In our study, to further understand the roles of *BBX* family genes in pear pollen growth, expression patterns of *PbBBX* genes were analyzed by transcriptome sequencing data. As shown in Figure (Additional file 3: Figure S2), 13 of *PbBBX* genes (52%) were not found to be expressed during the different developmental stages of pear pollen, implying that these genes might express in root, stem, leaf, or/and under special conditions. On the contrary, 12 *PbBBX* genes (48%) were detected to be expressed in a development-dependent pattern in pear pollen. For example, 5 *PbBBX*s (*PbBBX6*, *7*, *9*, *11*, *12*) were specifically expressed at P1 stage (mature pollen grains), while 2 (*PbBBX8* and *PbBBX10*) at P2 stage (hydrated pollen grains) (Additional file 3: Figure S2). The high number of *PbBBX* genes differentially expressed during pollen growth suggests that they are important proteins for signaling in this process. In addition, the expression patterns of *PbBBX* genes were validated by using qRT-PCR during pear pollen tube growth (Additional file 4: Figure S3). We found that qRT-PCR results were almost consistent with that of transcriptome sequencing data, except *PbBBX6* and *PbBBX7*. The reason for this divergence may be the lowest expression levels during pollen tube growth. Remarkably, compared with other periods, the expression levels of *PbBBX5* gene reached its peak at P4 (stopped-growth pollen tubes), implying that this gene might play potential role in plant reproductive development, such as the senescence of pollen tubes.

**Fig. 6:**
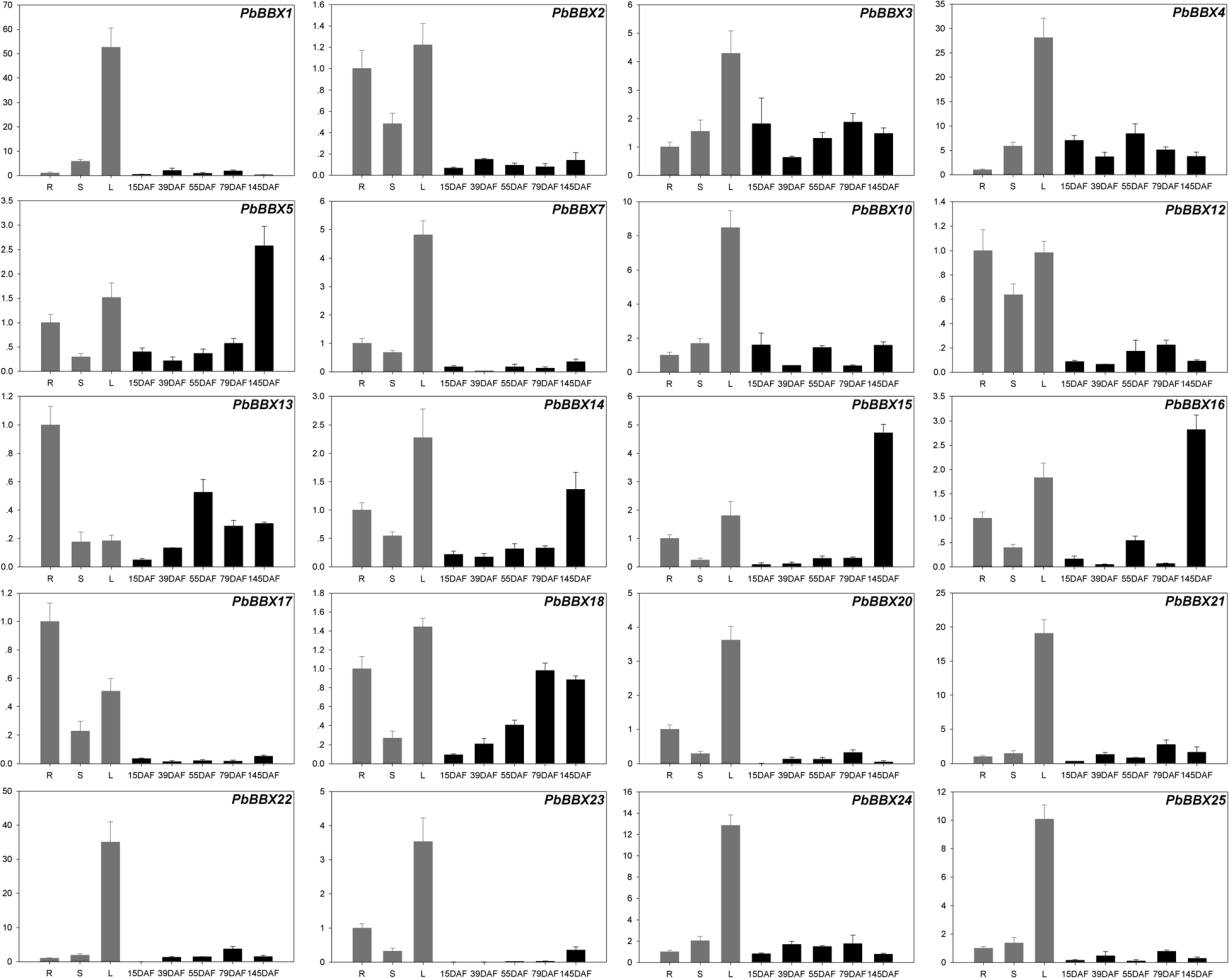
Expression levels of *PbBBX* genes in different plant tissues. 15 DAF (days after flowering), 39DAF, 55DAF, 79 DAF and 145 DAF correspond to five different developmental stages of pear fruit. In addition, root, stem and leaf were represented by R, S and L, respectively. The value on the left Y-axis indicates the relative gene expression levels

Subsequently, the expression profiles of these *PbBBX* genes in different tissues or organs were also surveyed by using qRT-PCR (Fig. 6). Results showed that except for *PbBBXs 6*, *8*, *9*, *11*, and *19* which showing no expression in the tested tissues or organs, *PbBBXs 1*, *2*, *3*, *4*, *7*, *10*, *14*, *16*, *18*, *20*, *21*, *22*, *23*, *24* and *25* were predominantly expressed in leaves, while *PbBBXs 13* and *17* were highly expressed in roots. Furthermore, the expression levels of *PbBBXs 12*, *17* and *25* was higher in root, stem or leaf than that those in different developmental stages of pear fruit. Interestingly, *PbBBXs 6*, *8*, *9*, and *11* were tissue-specifically expressed during different developmental stages of pear pollen. Additionally, we found *PbBBX19* was not expressed in root, stem, leaf and fruits, these results recommended that the functions need to be further studied at other tissues or special conditions (Fig. 6).

### Analysis of subcellular localization of *PbBBX4*, *PbBBX5* and *PbBBX13*

**Fig. 7:**
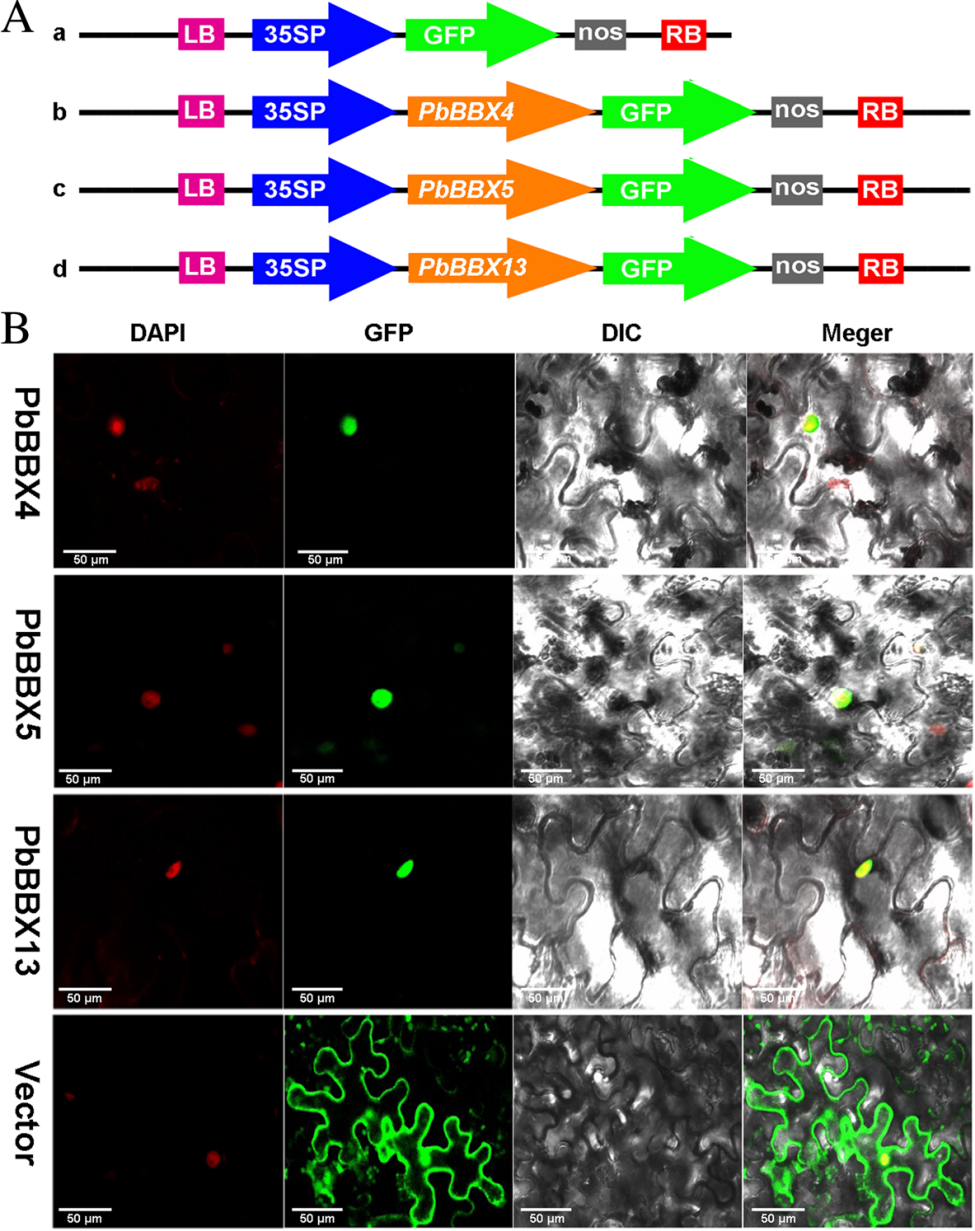
Subcellular localization of PbBBX4-GFP, PbBBX5-GFP and PbBBX13-GFP fusion protein. **a** Schematic representation of the 35S: GFP, 35S: PbBBX4-GFP, 35S: PbBBX5-GFP and 35S: PbBBX13-GFP fusion constructs used for transient expression. **b** The three PbBBX-GFP fusion proteins (PbBBX4-GFP, PbBBX5-GFP, and PbBBX13-GFP) as well as GFP as the control, were transiently expressed in tobacco leaves and observed by fluorescence microscopy. Bars = 50 μm

The nuclear localization of transcription factors is very important for its regulatory function. Previous studies reported that BBX proteins were predominantly located on the nucleus, such as SlBBX5, SlBBX7 and SlBBX15 in tomato [21]. The expression levels of *PbBBX4*, *PbBBX5* and *PbBBX13* observed that they play an important role in the development of pear pollen tubes or fruits. To further understand these three proteins characteristics, subcellular localization experiment was carried out. Subsequently, we introduced GFP control and the PbBBX4-GFP, PbBBX5-GFP and PbBBX13-GFP fusion constructs (Fig. 7a) by CaMV 35S promoter into *N. tabacum* epidermal cells. As indicated in Fig. 7b, green fluorescence signals from the expressed fusion PbBBX4-GFP, PbBBX5-GFP and PbBBX13-GFP were specifically distributed within the nuclei as confirmed by DAPI (DNA dye 4, 6-diamidino-2-phenylindole) staining. However, the control GFP protein was observed throughout the whole cell (Fig. 7b). These results suggested that PbBBX4, PbBBX5 and PbBBX13 were nuclear proteins, and consistent with the previous results [21].

## Discussion

Although the *BBX* gene family has been identified in several model plants, such as rice [22], and Arabidopsis [2], its function and evolution was still unclear in pear. In this study, a comprehensive analysis of pear *BBX* gene family was performed, including analyses of phylogeny, chromosome localization, gene duplication, sequence feature, and expression pattern.

A total of 25 *BBX* genes were identified from pear genome. The number of *BBX* genes in pear was fewer, compared to their orthologs in tomato (29) [21], Arabidopsis (32) [2], and rice (30) [22]. Noteworthy, the pear genome size (512 Mb) [17] was larger than those of rice (403 Mb) [23] or Arabidopsis (125 Mb) [24], although smaller than the tomato genome size (960 Mb) [25]. These results indicated that the *BBX* gene family members may not be directly related to the genome sizes in different plants. Although the difference in number was not significant, however, the type of *BBX* gene was different among species. In tomato, the numbers of BBX members two tandem B-BOXes plus the CCT domain, BOX1 plus CCT, two tandem B-BOXes, and B-BOX1 only were 8, 5, 10 and 6, respectively [21]. In Arabidopsis, the corresponding numbers were 13, 4, 8, and 7 [2]. And in pear were 7, 4, 9 and 6. These results indicated that *BBX* genes may have a common ancestor among different species, and were independently expanded after the divergence of the dicots and the monocots. In addition, to elucidate how the *BBX* gene family evolved, a phylogenic tree of plant *BBX* genes from monocots (rice and *Brachypodium distachyum*) and dicots (pear, poplar and Arabidopsis) was constructed. Within the phylogenic tree, *BBX*s were divided into five clades: I, II, III, VI, and V. We found that most of the *BBX* genes from the dicot were clustered together, implying that these genes might be orthologous genes as reported by previous studies [2, 18].

During the course of plant evolution, gene duplication plays an important role for generating novel genes. Gene duplication in plants has two main duplication patterns, including segmental duplication and tandem duplication [26], which had been demonstrated to play a key role in the expansion of gene family members in many species, such as the families *WOX*, *MYB*, *PRX* and *4CL* in pear [12, 27–29], the *CHS* in maize [30]. To further reveal the potential mechanism of evolution of the *BBX* gene family, both the segmental and tandem duplication events were analyzed in pear. In present study, none of the *PbBBX* genes were located in tandem. And 16 *PbBBX* genes were identified to be arranged in segmental duplication regions of pear chromosomes. These results indicated that segment duplications were the main driver force for the expansion of pear *BBX* gene family members. In addition, previous studies reported that tandem duplication often occurred in the large and rapidly evolving gene family, such as *NBS-LRR* gene family [31], whereas, segmental duplication usually occurred in the slowly evolving gene family, such as *MYB* gene family [31]. The present results indicated that pear *BBX* gene family should be classified as a gene family with slow evolutionary characteristics.

The collected transcriptome data showed that *B-BOX* gene family was involved in the pollen tube growth. Twelve of the pear *B-BOX* gene family members were found to be involved in the development of pear pollen tubes. The previous report by Gangappa et al. [32], has shown that during the photo-morphogenesis in Arabidopsis, some of BBX family members could competitively interact with protein and further regulate HY5 activity, leading to the fine regulation of the pollen tube development. Similarly, during the growth of pear pollen tube, the *BBX* family members might regulate the development process. Altogether, our results suggested that this gene family was not only involved in the development of floral organs, but also in the development of pollen tubes. The latter might be fulfilled by several genes (*PbBBX6*, *8*, *9*, and *11*) which were specifically expressed in pear pollen tubes. Previous studies by Gao et al. [20] suggested that as the extension of cultural duration to 15 h (P4: stopped-growth pollen tubes), pear pollen tubes growth became slow and exhibited some characteristics of senescence at P4 post-cultured in vitro, implying that the senescence of the pear pollen tubes might occur at the P4 period. In the present study, the expression level of *PbBBX5* in the pear pollen tubes was significantly increased, and its expression pattern was basically consistent with the previous report [20]. The increased expression level of *PbBBX5* suggested that it might play a role in the regulation of pollen tube senescence.

Additional, the expression patterns of both *PbBBX4* and *PbBBX13*, were consistent with the content dynamics of fruit lignin: at the early to middle stages the concentration of these contents increased, while at the mature stage showed less concentration [27, 33, 34]. These results suggested that these two genes might regulate the lignin synthesis in pear fruits. As reported by our previous studies, the content of stone cells was thought to be an important factor affecting the quality of pear fruit [33, 34]. Due to closely relationship between the development of stone cell and the biosynthesis of lignin [33, 34], it is possible that *PbBBX4* and *PbBBX13* could be applied for the improvement of pear fruits using genetic engineering.

## Conclusions

In the present study, a systematic analysis of the *PbBBX* gene family was carried out, including conserved domain, gene structure, phylogenetic relationship, chromosome location, gene duplication and expression pattern analysis. The *PbBBX* genes were divided into five clades: I (4 genes), II (4 genes), III (3 genes), IV (9 genes), V (5 genes), which were supported by gene structural and conserved domain analysis. Gene duplication analysis suggested that the segmental duplications have driven expansion of the pear *BBX* gene family. Transcriptome sequencing and qRT-PCR analysis revealed that the *PbBBX* genes play an important role in different pollen tube and fruit developmental stages. Further analysis revealed that *PbBBX4* and *PbBBX13* might regulate the synthesis of pear fruit lignin, and *PbBBX5* might play a role in the senescence of pollen tubes.

## Methods

### Sequence retrieval

To identify and annotate *BBX* genes in pear, the Arabidopsis BBX protein sequences [2] from the Arabidopsis Information Resource (TAIR) database (http://www.arabidopsis.org) were used as queries to search against pear genome database with BLASTP program (e-value <1e-5). Subsequently, the putative *BBX* genes in pear genome, were verified for the presence of the B-BOX domain by screening against the InterProScan (http://www.ebi.ac.uk/Tools/pfa/iprscan/) [35], Pfam (http://pfam.sanger.ac.uk/) [36] and SMART (http://smart.embl-heidelberg.de/) [37] database.

### Phylogenetic analysis and sequence alignment

Multiple sequence alignments of the 25 pear BBX proteins were generated using ClustalW version 1.83 with default settings, and the neighbor-joining (NJ) tree was constructed by MEGA 5.2 with bootstrap analysis (1000 replicates) [38]. The pfam (http://pfam.xfam.org) [36], InterProscan (http://www.ebi.ac.uk/interpro/scan.html) [35], and SMART (http://smart.embl-heidelberg.de) [37] were used to identify domains. The sequence logos of conserved domains were generated using online WebLogo (http://weblogo.berkeley.edu/logo.cgi) [39].

### Gene structure, chromosomal location, and duplication analysis

The exons and introns of the *BBX* genes were identified according to the pear genome annotation file. And exon-intron map was generated by using Gene Structure Display Server (http://gsds.cbi.pku.edu.cn/) [40]. The chromosome location image of the pear *BBX* genes on chromosomes or scaffolds was drawn using the MapInspect software according to the physical positions on the pear chromosomes. The MCScanX software (http://chibba.pgml.uga.edu/mcscan2/) [41] was used to identify the duplications of *PbPRXs*. The Calculator 2.0 software [42] was used to estimate the nonsynonymous (Ka) and synonymous (Ks) substitution rates of the different gene duplication pairs. The Ks values were used to estimate the approximate date of every duplicated event occurred in pear, seeing the formula: T = Ks/2λ × 10^–﻿6﻿^ Mya (λ =6.5 × 10^−9^) [17, 43].

### Plant material

The samples were collected from ten of healthy, 40-year-old pear trees (*Pyrus bretschneideri cv*. Dangshan Su), which have been managed under the same irrigation and fertilization in the orchard at Dangshan County, Anhui province, China. These pear samples under the same developmental period were grown toward the middle southern direction and collected on early April, 2016. Roots, stems, and leaves were collected at the fifteen the day after flowering (DAF). 40 fruits with the uniform size were collected on 19th April (15 DAF), 14 May (39 DAF), 30 May (55 DAF), 22 June (79 DAF), and 29 August (145 DAF) in 2016, respectively. The methods for collection and drying, and in vitro culture of pear pollen grains, were based on the procedures by Zhou. et al. 2016 [44].

### RNA-seq expression analysis

The raw RNA-seq reads from pear pollen were download from the NCBI database (PRJNA299117) [44]. The pear pollen samples were as follows: P1: mature pollen grains, P2: hydrated pollen grains, P3: growing pollen tubes, and P4: stopped-growth pollen tubes. The analysis of raw RNA-seq data was according to previous method [45], and the RPKM (Reads Per Kilobase per Million mapped reads) values were used to estimate the gene expression level. The heatmap of *PbBBX* genes was exhibited using R software (http://www.bioconductor.org/).

### qRT-PCR analysis

The TIANGEN RNAprep pure (Tiangen, Beijing, China) was used to extract the total RNA according to the manufacturer’s instructions, followed by DNaseI (Tiangen, Beijing, China) digestion to eliminate any contaminating DNA. For qRT-PCR analysis, the first-strand cDNAs was synthesized from the 1 μg RNA using the Reverse Transcriptase M-MLV System (Tiangen, Beijing, China) according to the manufacturer’s instructions. The Beacon Designer 7 software was used to design and check the gene-specific primers (Additional file 5: Table S2). The pear tubulin gene (forward primer: 5′ -AGAACAAGAACTCGTCCTAC-3′; reverse peimer: 5′-GAACTGCTCGCTCACTCTCC-3′) was used as reference gene [46]. The qRT-PCR was carried out using SYBR® Premixm Ex Taq™ (TaKaRa, Japan) with the CFX96 Touch™ Real-Time PCR Detection System (Bio-Rad, USA). For each sample, we executed three biological replicates. The 2 ^–ΔΔCT^ method was used to estimate the relative expression level [47].

### Subcellular localization analysis

The expression vectors of 35S:PbBBX5-GFP, 35S: PbBBX4-GFP, and PbBBX13-GFP were constructed by insertion of cDNA *PbBBX5*, *PbBBX4* and *PbBBX13*, into pCAMBIA1304 vector, respectively. After electroporation of these construction into *Agrobacterium tumefaciens* EHA105, using pCAMBIA1304 vector as negative control [48], the transformed bacterial cells were infected into the leaf tissue of *Nicotiana tabacum* as the method described by Sparkes et al. (2006) [49]. The transient expression of PbBBX-GFP was observed using a laser confocal microscope (Zeiss LSM700, Germany), the DNA dye 4,6-diamidino-2-phenylindole (DAPI) was used to visualize the nucleus.

## Additional files

Additional file 1: Figure S1. Chromosomal locations and duplication events of *BBX* genes in the pear genome. The chromosome number is implied at the top of each chromosome. The segmental duplicated genes are connected by color lines and marked by corresponding color boxes. The scale on the left represents the megabases (Mb). (TIFF 3910 kb)

Additional file 2: Table S1. Ka/Ks analysis and divergence time estimated for pear duplicated *BBX* paralogs. (XLSX 11 kb)

Additional file 3: Figure S2. Expression profiles of *PbBBX* genes in pear pollen. P1, P2, P3, and P4 are represents mature dry pollen, hydrated pollen grains, pollen tubes, and stop growing pollen tubes, respectively. Color scale represents log2 transformed RPKM (Reads Per Kilobase per Million mapped reads) values. The gradually change of the color indicates different expression level of *PbBBX* genes, and the middle expression level was represented by white color. (TIFF 87 kb)

Additional file 4: Figure S3. The gene expression of 12 *PbBBX* genes during pollen tube growth by qRT-PCR. The value on the left Y-axis indicates the relative gene expression levels. P1 (mature pollen grains), P2 (hydrated pollen grains), P3 (growing pollen tubes), and P4 (stopped-growth pollen tubes) correspond to four different developmental stages of pollen and pollen tube. (TIFF 123 kb)

Additional file 5: Table S2. Primers in this study. (XLSX 11 kb)

## Abbreviations

BBX: B-BOX
COP1: Constitutively Photomorphogenic 1
DAF: Days after flowering
DAPI: DNA dye 4,6-diamidino-2-phenylindole
HMM: Hidden Markov Model
HY5: Protein long hypocotyl 5
Ka: Nonsynonymous
Ks: Synonymous
MEME: Multiple Em for Motif Elicitation
Mya: Million years
NJ: Neighbor joining
qRT-PCR: Real-Time PCR
RPKM: Reads Per Kilobase per Million mapped reads
