# Supplementary figures and images for: B-BOX genes: genome-wide identification, evolution and their contribution to pollen growth in pear (Pyrus bretschneideri Rehd.)
Source: BMC Plant Biol. 2017 Sep 19;17:156. doi: 10.1186/s12870-017-1105-4 (PMC5606111; doi:10.1186/s12870-017-1105-4)

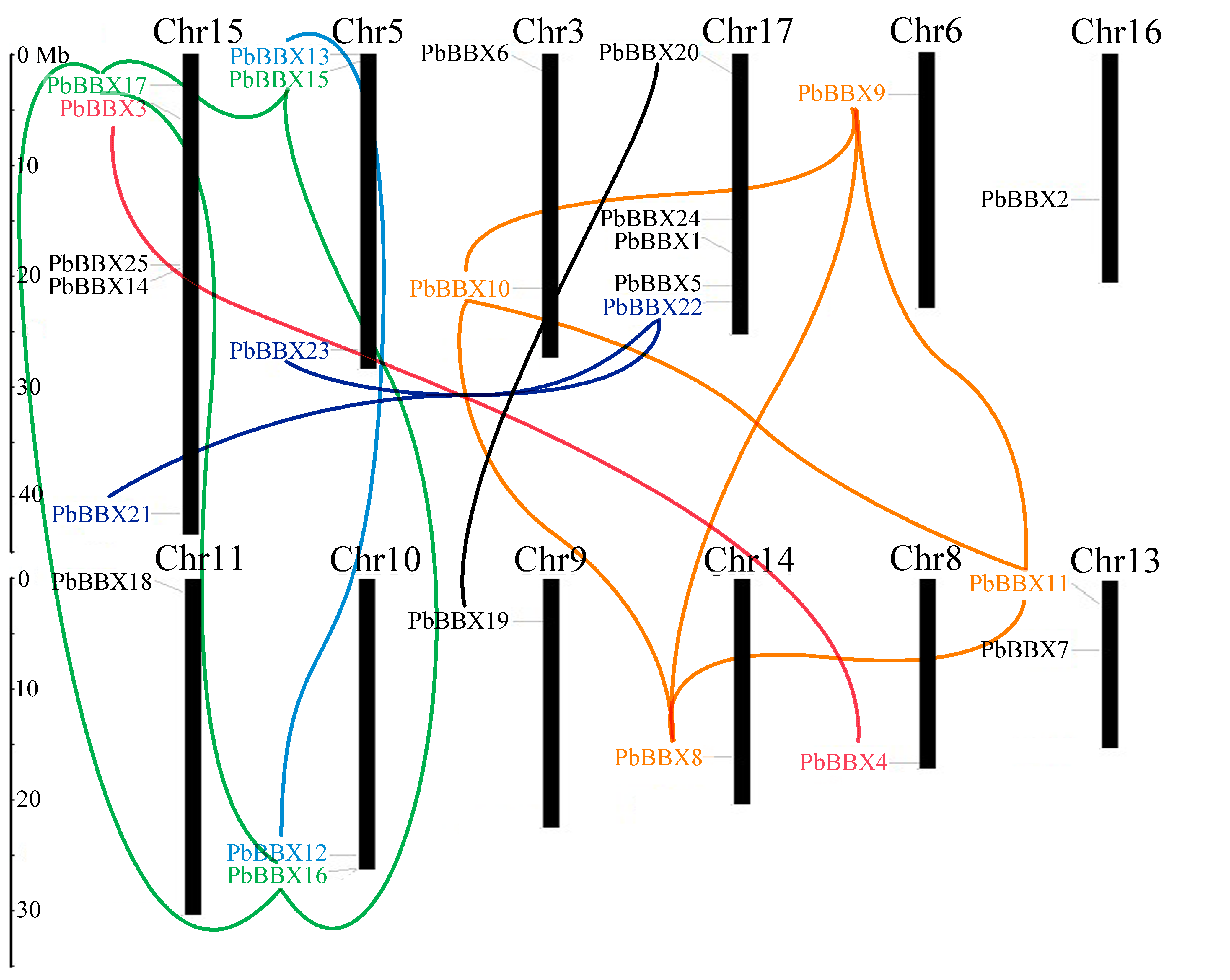

Supplement: Supplementary file 1 — Chromosomal locations and duplication events of BBX genes in the pear genome. The chromosome number is implied at the top of each chromosome. The segmental duplicated genes are connected by color lines and marked by corresponding color boxes. The scale on the left represents the megabases (Mb). (TIFF 3910 kb) [file 12870_2017_1105_MOESM1_ESM.tif]

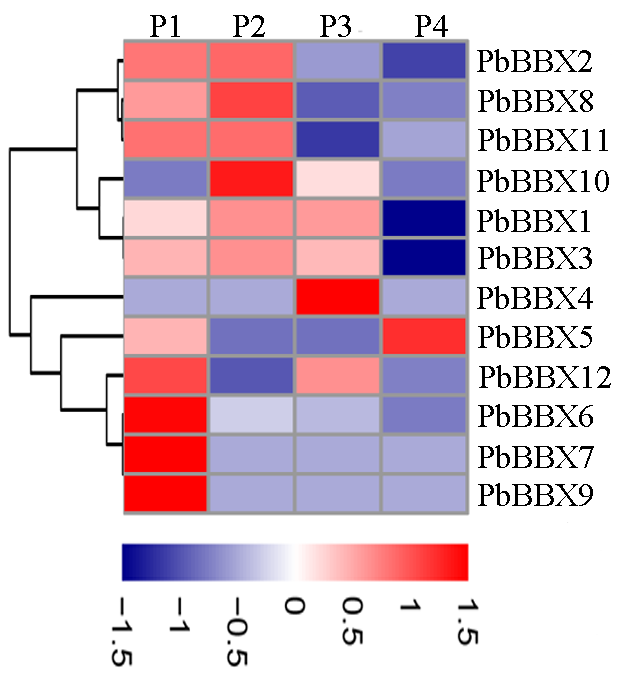

Supplement: Supplementary file 3 — Expression profiles of PbBBX genes in pear pollen. P1, P2, P3, and P4 are represents mature dry pollen, hydrated pollen grains, pollen tubes, and stop growing pollen tubes, respectively. Color scale represents log2 transformed RPKM (Reads Per Kilobase per Million mapped reads) values. The gradually change of the color indicates different expression level of PbBBX genes, and the middle expression level was represented by white color. (TIFF 87 kb) [file 12870_2017_1105_MOESM3_ESM.tif]

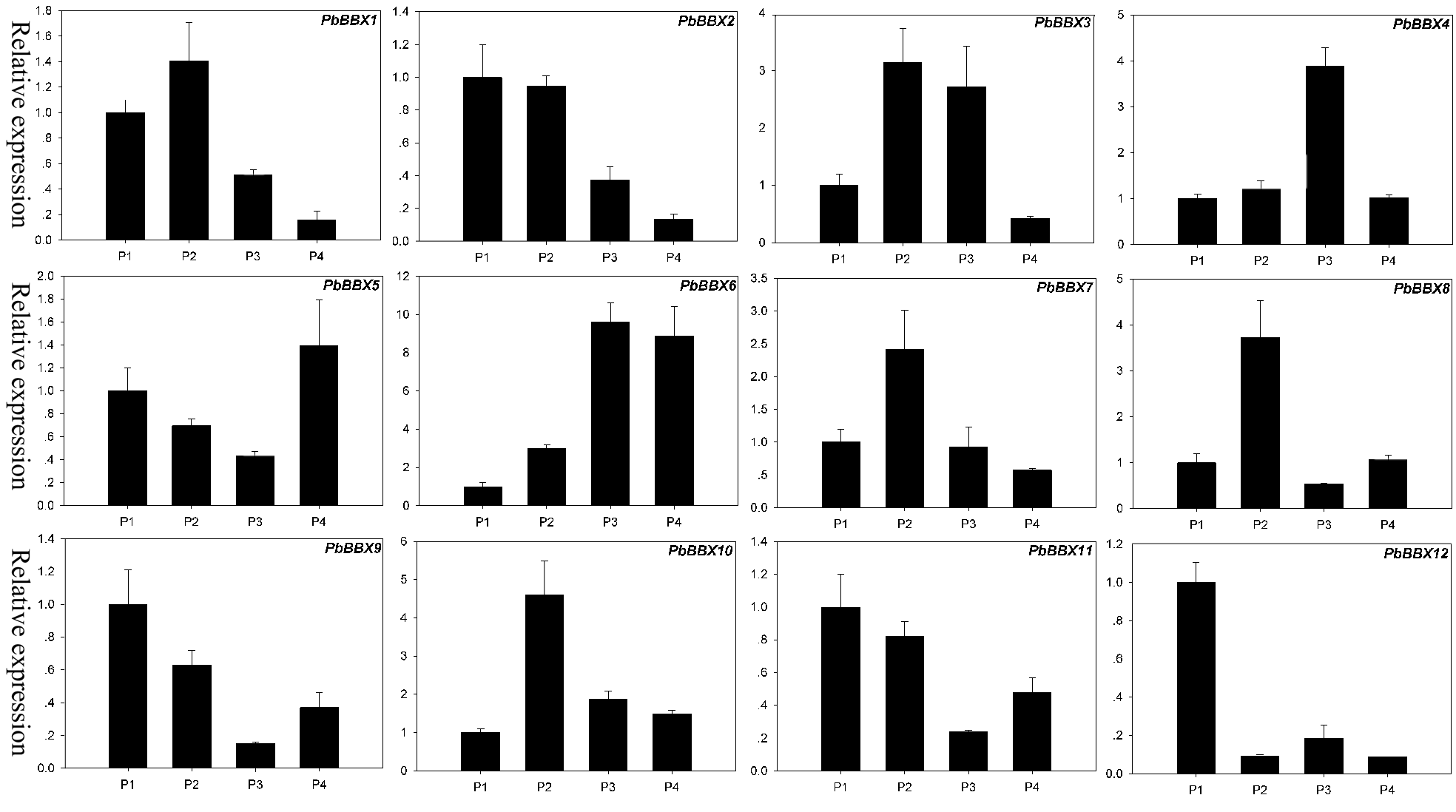

Supplement: Supplementary file 4 — The gene expression of 12 PbBBX genes during pollen tube growth by qRT-PCR. The value on the left Y-axis indicates the relative gene expression levels. P1 (mature pollen grains), P2 (hydrated pollen grains), P3 (growing pollen tubes), and P4 (stopped-growth pollen tubes) correspond to four different developmental stages of pollen and pollen tube. (TIFF 123 kb) [file 12870_2017_1105_MOESM4_ESM.tif]
